# Age-related differences in intrinsic neural timescales (INTs): an analysis of autocorrelation windows (ACWs) in resting-state EEG across young and older adults

**DOI:** 10.1186/s12883-025-04491-z

**Published:** 2025-11-21

**Authors:** Shannon R. Hayashi, Ani Grigoryan, Tom Froese

**Affiliations:** 1 Embodied Cognitive Science Unit, Okinawa Institute of Science and Technology Graduate University, Onna, Okinawa Japan; 2 Research Laboratory of Personality and Social Environment, Faculty of Philosophy and Psychology, Yerevan State University, Yerevan, Armenia

**Keywords:** Aging, Autocorrelation windows, Electroencephalography, Resting-state, Temporal integration, Intrinsic neural timescales, Brain aging, Lifespan development

## Abstract

**Background:**

Autocorrelation windows (ACWs) quantify the temporal continuity of neural activity and have been linked to self-related processing and conscious experiences. Research suggests that longer ACWs during the resting state reflect a brain’s capacity to maintain temporal integration over extended periods. However, the influence of aging on these fundamental intrinsic neural timescales (INTs) remains largely unexplored, despite extensive evidence of age-related changes in brain structure and function.

**Methods:**

We analyzed resting-state EEG data from 196 healthy adults (137 younger: 20–35 years; 59 older: 59–77 years) from the Leipzig Study for Mind-Body-Emotion Interactions (LEMON) dataset. Three ACW measures were calculated across multiple electrode selection strategies: ACW-0, ACW-e, and ACW-50. Statistical analyses included parametric and non-parametric group comparisons, principal component analysis, and hierarchical modeling approaches with comprehensive methodological controls.

**Results:**

Older adults demonstrated consistently shorter ACWs than younger adults (Cohen’s *d* range: -0.33 to -0.48, all *p* < 0.01), indicating decreased temporal continuity in neural activity with age. Parametric and non-parametric tests confirmed these effects. This effect was robust across multiple electrode selection criteria and represented a global brain-wide phenomenon (the first principal component explained 55–70% of the variance).

**Conclusions:**

These findings provide empirical evidence for robust age group differences in intrinsic neural timescales (INTs). Results demonstrate that older adults exhibit systematically shorter neural timescales compared to younger adults, with implications for understanding age-related changes in neural network organization.

## Background

Understanding how the brain maintains its functional capacity across the lifespan represents a fundamental challenge in neuroscience with direct implications for healthy aging and cognitive reserve. Brain maintenance refers to the biological processes that preserve neural health despite age-related structural changes, while cognitive reserve encompasses the mental resources that enable individuals to cope with brain aging and pathology [1, 2]. Recent advances in neuroscience have revealed that the brain’s capacity for temporal integration may serve as a marker of brain maintenance and cognitive reserve [3, 4]. This may be particularly evident in cortical midline structures, which support self-referential processing [5] and have been linked to temporal integration capacity.

Autocorrelation windows (ACWs) quantify the degree of temporal continuity in neural activity by measuring how the brain’s activity at one point relates to its activity at subsequent time points [4, 6–9]. Northoff et al. demonstrated that ACWs provide an empirical assessment of the brain’s intrinsic neural timescales (INTs), with longer ACWs indicating sustained temporal integration capacity and shorter ACWs reflecting rapid decorrelation of neural signals [7, 10]. These temporal integration processes have been validated across multiple cognitive domains and linked to individual differences in behavioral outcomes [3, 9]. Notably, ACWs were longer during cognitively demanding tasks than during resting states, suggesting that temporal integration capacity underlies various cognitive processes including attention and memory consolidation [11, 12]. This relationship appears particularly strong for self-related processing: participants showed longer autocorrelation windows during self-narrative tasks compared with non-self conditions [11]. Moreover, INTs in cortical midline structures and prefrontal regions mediated cognitive bias in self-related decision-making tasks [12]. Alterations in INTs occur in various neurological and psychiatric conditions, suggesting that ACW measures serve as sensitive indicators of neural system integrity [4, 7, 8].

Brain aging involves complex structural and functional changes including gray matter volume reduction, white matter integrity decline, and altered connectivity patterns, particularly in large-scale networks [2, 13, 14]. Despite these changes, the aging brain demonstrates capacity for reorganization through neuroplasticity, with compensatory recruitment of neural resources and modified processing strategies that help maintain cognitive performance. This interplay between decline and adaptation raises fundamental questions about how aging processes affect temporal integration capacity. If INTs serve as markers of neural efficiency and cognitive reserve, understanding how ACWs change with age and how they relate to adaptive mechanisms could provide insights into successful brain maintenance in older adults.

Previous research on INTs has predominantly focused on young adult populations, with limited systematic investigation of age-related changes in temporal integration capacity. This represents a significant gap, particularly given evidence linking disrupted temporal processing to age-related cognitive changes and neurological conditions. Recent advances in EEG-based ACW measurements provide opportunities to examine INTs across broad age ranges in large samples.

We address this gap by leveraging the Leipzig Study for Mind-Body-Emotion Interactions (LEMON) dataset [15] to examine age-related differences in INTs. Given the evidence for age-related alterations in neural structure and function, including reductions in neural efficiency and connectivity, we hypothesized that older adults would exhibit shorter ACWs than younger adults.

## Methods

### Data analysis

We conducted a comprehensive analysis of age-related differences in ACW measures from resting-state EEG data, incorporating both descriptive statistics and hierarchical mixed-effects modeling to account for the nested data structure.

### Participants and age grouping

The original LEMON dataset [15] included 227 participants (153 young adults and 74 older adults). After the LEMON’s EEG preprocessing pipeline, 203 participants were available for analysis. We categorized participants into Young (20–35 years) and Older (59–77 years) adults, consistent with the original LEMON study design treating age as a categorical variable. Seven participants fell outside these pre-established age ranges (four middle-aged and three exceeding the upper limit) and were excluded following the original LEMON age criteria. The final analysis sample included 137 young adults and 59 older adults (*n* = 196 total).

### EEG data collection and preprocessing

We utilized the publicly available preprocessed resting-state EEG data from LEMON. This dataset was selected for this investigation because it provides high-quality resting-state EEG data from a large sample spanning a broad age range (20–77 years), making it ideal for examining age-related changes in ACWs while providing sufficient statistical power for categorical age comparisons.

#### EEG recording parameters

The original LEMON resting-state EEG protocol recorded 16-minute sessions using a 62-channel BrainAmp MR plus amplifier with active ActiCAP electrodes positioned according to the international 10–20 extended system, referenced to FCz. Data were acquired at 2500 Hz with a bandpass filter between 0.015 Hz and 1 kHz. Sessions comprised alternating 60-second blocks of eyes-closed and eyes-open conditions (eight blocks each), with participants seated in an electrically shielded booth.

#### LEMON preprocessing pipeline

The LEMON team applied a standardized preprocessing pipeline including, (1) downsampling to 250 Hz, (2) bandpass filtering (1–45 Hz, 8th order Butterworth), (3) separation into eyes-closed and eyes-open conditions, (4) visual inspection and removal of poor-quality channels and data segments, (5) principal component analysis retaining components explaining 95% of variance, (6) independent component analysis (ICA) using the Infomax algorithm for artifact removal, and (7) back-projection of retained components to sensor space.

In the present study, we utilized eyes-closed condition data, as this represents the most standardized resting-state condition for examining INTs without external visual input.

### Quality control and segment selection

The preprocessed eyes-closed resting-state data from the LEMON dataset were segmented into epochs ranging from 1 to 60 s (mean = 56.2s), with participants having 8–15 segments. To ensure data quality and standardization, segments were filtered based on the duration criteria (55–65 s, targeting 60-second epochs).

### Statistical analysis

#### Electrode selection strategies

We analyzed the data using three electrode selection strategies: all available electrodes (discovery approach), electrodes present in ≥ 95% of segments (balanced approach), and electrodes present in 100% of segments (conservative approach).

#### Primary group comparisons

For each ACW measure (ACW_0, ACW_e, ACW_50), we conducted participant-level comparisons using Welch’s *t*-tests and Mann-Whitney *U* tests, comparing young and older groups using data averaged across all segments and channels per participant. Effect sizes were calculated using Cohen’s *d* with 95% confidence intervals using the pooled standard deviation method.

### Multi-level analyses

#### Channel-wise analysis

Individual electrode comparisons between age groups were performed using participant-averaged data across 61 electrodes, with False Discovery Rate (FDR) correction using the Benjamini-Hochberg method to control for multiple comparisons.

#### Regional analysis

For descriptive analyses, channels were grouped into eight anatomical regions (Frontal, Central, Parietal, Occipital, Temporal, Fronto-Central, Centro-Parietal, Parieto-Occipital). For mixed-effects modeling, channels were grouped into five primary anatomical regions (Frontal, Central, Parietal, Occipital, Temporal) to improve the statistical power and model convergence.

#### Principal component analysis

To identify global vs. regionally-specific age effects, we performed PCA on participant × channel matrices for each ACW measure. Age group differences were tested on the first five principal components using *t*-tests.

#### Within-group age associations

Pearson and Spearman correlations between continuous age and ACW measures were calculated separately within Young and Older groups, with FDR correction applied within each age group.

### Hierarchical mixed-effects modeling

To properly account for the nested data structure (channels within segments nested in participants), we implemented mixed-effects models using variance decomposition to guide model selection. We calculated intraclass correlations (ICCs) at the participant and segment levels to determine appropriate model structure. When segment-level variance accounted for < 5% of total variance, we used 2-level models; when ≥ 5%, we used 3-level models. In cases where convergence issues arose with complex nested structures, temporal stability was assessed using direct correlation analyses between segment position and ACW measures rather than hierarchical modeling approaches.

Models were fitted using the statsmodels.mixedlm function with the general form:

- 2-level model: ACW ~ Age_Group_Older + Segment_Position_Normalized + (1|Participant_ID)
- 3-level model: ACW ~ Age_Group_Older + Segment_Position_Normalized + (1|Participant_ID)+ (1|Participant_ID:Segment_Number

Age_Group_Older was coded as a binary variable (0 = Young, 1 = Older) and Segment_Position_Normalized was included as a covariate to control for temporal order effects within sessions.

### Variance decomposition and model selection strategy

Prior to the primary analyses, variance decomposition was conducted for each ACW measure to determine the appropriate hierarchical structure and to guide the analytical approach. Intraclass correlation coefficients (ICCs) were calculated to assess the proportion of variance at participant and segment levels. When segment-level variance accounted for < 5% of total variance, 2-level models were used; when ≥ 5%, 3-level models were implemented, consistent with statistical best practices for nested data structures.

### Methodological controls and diagnostics

#### Temporal controls

Segment position effects were controlled through normalization of segment order within each participant (z-score transformation), addressing potential temporal order effects within recording sessions.

#### Missing data strategy

Three electrode selection strategies were employed to address data completeness and ensure robustness: (1) all available electrodes (61 channels, discovery approach), (2) electrodes present in ≥ 95% of valid segments (57 channels, empirical 95%), and (3) electrodes present in 100% of valid segments (21 channels, empirical 100%, most conservative approach).

#### Outlier detection

IQR-based outlier detection (1.5 × IQR criterion) was implemented across all ACW measures, with outlier warnings generated for quality assessment without exclusion of data points.

#### Temporal stability validation

To ensure that age-related differences were not confounded by temporal order effects, relationships between normalized segment position and ACW measures were examined using both Pearson and Spearman correlations across all electrode selection strategies.

We implemented cross-level validation examining effect size consistency across participant-level, segment-level, and channel-level aggregations. For mixed-effects models, we evaluated model convergence using BFGS optimization, model fit indices (AIC, BIC, log-likelihood), residual normality using Shapiro-Wilk tests, and random effects variance estimates. When models failed to converge (primarily at individual channel levels), we implemented regional aggregation approaches to improve statistical power.

#### Statistical software and significance criteria

All analyses were conducted in Python 3.7.3 using scipy.stats, statsmodels, and sklearn libraries. Statistical significance was set at *α* = 0.05, with FDR-corrected *p*-values reported for multiple comparisons. Effect sizes were interpreted using Cohen’s conventions (small: *d* ≥ 0.2, medium: *d* ≥ 0.5, large: *d* ≥ 0.8). This comprehensive analytical approach ensured robust inference while accounting for the complex nested structure inherent in multi-channel, multi-segment EEG data.

### Figure preparation

Publication-quality figures were generated using Python 3.7.3 with matplotlib and seaborn. Topographic maps were created using MNE-Python with standard 10–20 electrode positioning

### AI Assistance

Claude.ai assisted with Python code implementation of methodological revisions for statistical analysis and visualization. All codes were reviewed and validated by the authors.

## Results

### Variance decomposition and hierarchical structure

Intraclass correlation coefficients revealed that segment-level variance consistently accounted for 0.9–1.3% of total variance across ACW measures (ACW_0: 0.9%, ACW_e: 0.9%, ACW_50: 1.3%). In contrast, participant-level variance explained 16.7–30.7% of total variance depending on the measure (ACW_0: 24.0%, ACW_e: 30.7%, ACW_50: 16.7%). Given that all segment-level variance was below the 5% threshold, we implemented a simplified analytical approach focusing on participant-level differences rather than complex three-level hierarchical modeling.

### Temporal stability validation

No significant associations were detected between segment position and any ACW measure (18 correlations total: Pearson *r* range = −0.007 to 0.014, Spearman *ρ* range = −0.006 to 0.011; all *p* ≥ 0.594). These findings confirm temporal stability of ACW measures throughout recording sessions and validate that observed age differences represent genuine neurobiological phenomena rather than temporal artifacts.

### Primary age effects on ACWs

#### Parametric and non-parametric group comparisons

**Fig. 1:**
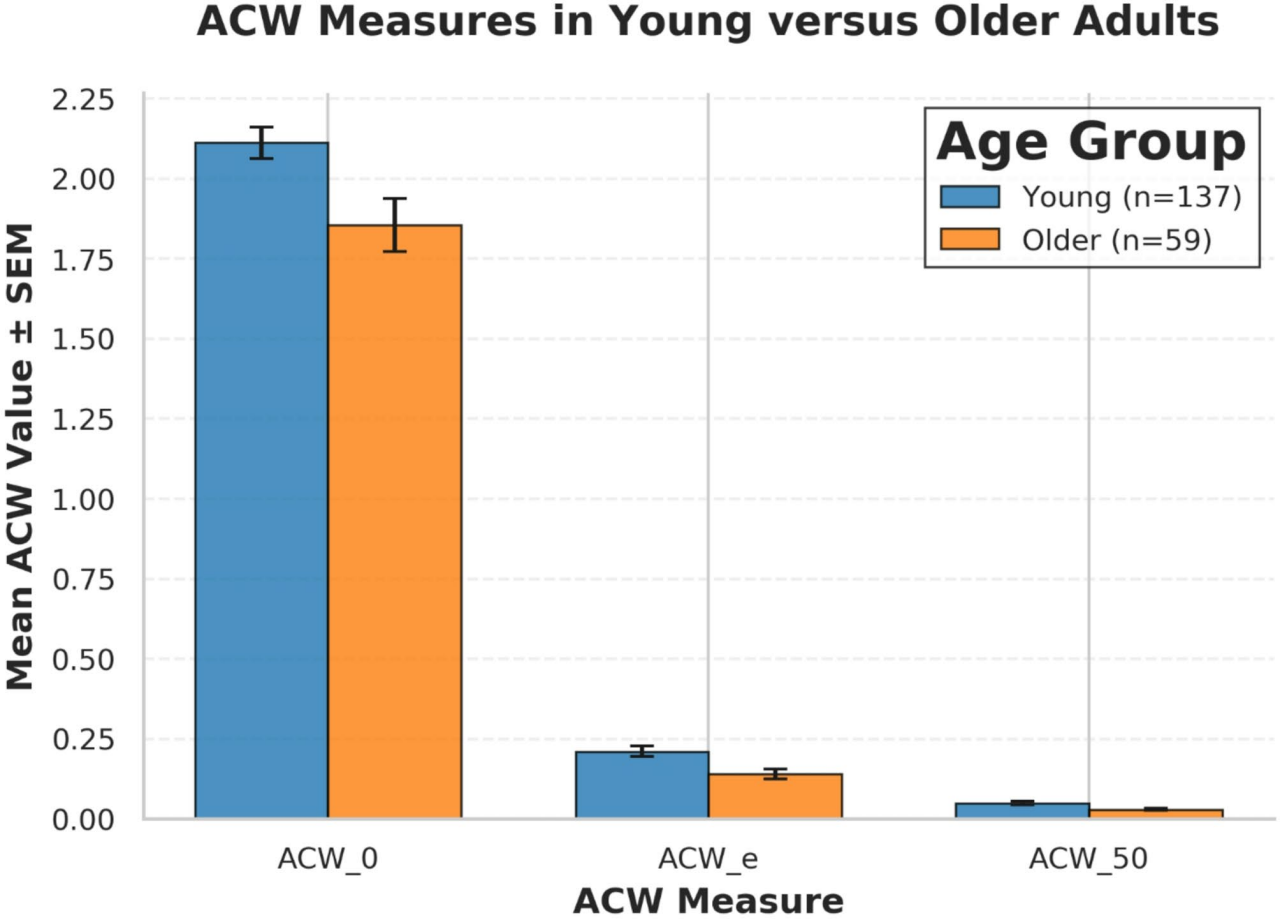
ACW measures in young versus older adults. Bar plot showing mean (± *SEM*) ACW values for young (*n* = 137) and older (*n* = 59) adults across three temporal measures. ACW_0 represents zero-lag autocorrelations, ACW_e represents extended-scale temporal integration, and ACW_50 represents intermediate-range temporal dynamics. Error bars indicate standard error of the mean. All measures showed significant age-related decreases (all *p* < 0.01, Cohen’s *d* range = −0.328 to −0.480)

Using the all electrodes strategy (61 channels), young adults demonstrated significantly higher ACW values than older adults across all three temporal measures. Age-related differences in ACW measures are illustrated in Fig. 1. Both parametric (Welch’s *t*-test) and non-parametric (Mann-Whitney *U*) tests yielded consistent results.

#### ACW_0 (Zero-lag autocorrelation window)

- Young adults: M = 2.112, 95% CI [2.016, 2.207], *n* = 137
- Older adults: M = 1.855, 95% CI [1.690, 2.020], *n* = 59
- Mean difference: −0.257 (Older - Young)
- Welch’s *t* = 2.69, *p* = 0.008
- Mann-Whitney *U* = 5,443, *p* = 0.0001
- Cohen’s *d* = −0.438, 95% CI [−0.748, −0.128] (small to medium effect)

#### ACW_e (1/e threshold autocorrelation window)

- Young adults: M = 0.211, 95% CI [0.178, 0.244], *n* = 137
- Older adults: M = 0.140, 95% CI [0.109, 0.172], *n* = 59
- Mean difference: −0.071 (Older - Young)
- Welch’s *t* = 3.08, *p* = 0.002
- Mann-Whitney *U* = 4,904, *p* = 0.018
- Cohen’s *d* = −0.399, 95% CI [−0.709, −0.089] (small to medium effect)

#### ACW_50 (50% threshold autocorrelation window)

- Young adults: M = 0.049, 95% CI [0.039, 0.059], *n* = 137
- Older adults: M = 0.030, 95% CI [0.022, 0.038], *n* = 59
- Mean difference: −0.019 (Older - Young)
- Welch’s *t* = 2.98, *p* = 0.003
- Mann-Whitney *U* = 5,206, *p* = 0.001
- Cohen’s *d* = −0.371, 95% CI [−0.681, −0.062] (small to medium effect)

All effect sizes indicated small to medium magnitude differences according to Cohen’s conventions, with negative values reflecting higher ACW values in young compared to older adults, consistent with shorter neural timescales in aging.

**Fig. 2:**
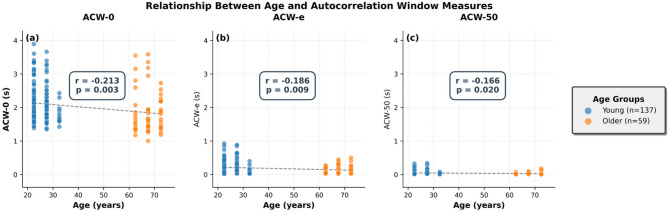
Relationship between age and autocorrelation window measures. Scatterplots showing individual participant data for (**a**) ACW-0: zero-crossing threshold, (**b**) ACW-e: 1/*e* decay threshold, and (**c**) ACW-50: 50% decay threshold. Young (20–35 years, *n* = 137, blue) and Older (59–77 years, *n* = 59, orange) adults from the LEMON dataset are shown with black dashed lines representing linear regression fits across all participants. Pearson correlation coefficients and p-values are displayed in the center of each panel. Data represent 60-electrode EEG recordings during eyes-closed resting state

To examine the continuous relationship between age and ACW measures across all participants, Fig. 2 presents scatterplots showing individual data points. These plots reveal the categorical study design with middle-aged participants (36–58 years) excluded, creating a 22-year gap between groups. While significant negative correlations exist between age and all ACW measures across the full sample (see statistics in the figure), the regression lines illustrate that this relationship appears to be driven primarily by group differences rather than continuous gradients within groups.

### Cross-strategy validation and robustness

To establish robustness across varying data completeness criteria, age effects were systematically validated using increasingly conservative electrode selection approaches. The effect sizes remained remarkably consistent across strategies:

#### Cross-strategy effect size validation with dual statistical confirmation

**Table:** 

Strategy	ACW_0	ACW_e	ACW_50
All Electrodes (61 channels)	*d* = −0.438, 95% CI [−0.748, −0.128]	*d* = −0.399, 95% CI [−0.709, −0.089]	*d* = −0.371, 95% CI [−0.681, −0.062]
	*t*-test: *p* = 0.008**	*t*-test: *p* = 0.002**	*t*-test: *p* = 0.003**
	Mann-Whitney: *p* = 0.0001**	Mann-Whitney: *p* = 0.018*	Mann-Whitney: *p* = 0.001**
Empirical 95% (57 channels)	*d* = −0.456, 95% CI [−0.766, −0.145]	*d* = −0.405, 95% CI [−0.714, −0.095]	*d* = −0.372, 95% CI [−0.681, −0.063]
	*t*-test: *p* = 0.006**	*t*-test: *p* = 0.002**	*t*-test: *p* = 0.003**
	Mann-Whitney: *p* < 0.0001**	Mann-Whitney: *p* = 0.014*	Mann-Whitney: *p* = 0.001**
Empirical 100% (21 channels)	*d* = −0.480, 95% CI [−0.791, −0.169]	*d* = −0.381, 95% CI [−0.690, −0.071]	*d* = −0.328, 95% CI [−0.637, −0.019]
	*t*-test: *p* = 0.004**	*t*-test: *p* = 0.005**	*t*-test: *p* = 0.014*
	Mann-Whitney: *p* < 0.0001**	Mann-Whitney: *p* = 0.019*	Mann-Whitney: *p* = 0.001**

*p < 0.05, **p < 0.01

Effect size concordance across strategies was exceptional, with maximum differences of only 0.044 Cohen’s *d* units for any given measure (ACW_50 range: −0.328 to −0.372), demonstrating that the findings were not dependent on electrode inclusion criteria. Critically, the most conservative approach (empirical 100%) maintained statistical significance across all measures despite reduction in spatial coverage from 61 to 21 channels, confirming robustness against missing data artifacts.

### Spatial distribution and topographic organization

#### Regional analysis across anatomical territories

Age-related differences exhibited systematic spatial organization across eight anatomical regions. Regional analysis revealed effect sizes ranging from small to medium (*d* = −0.266 to −0.540).

####  ACW_0 showed the strongest regional differentiation

- Centro-Parietal: *d* = −0.521, *t*-test *p* = 0.0008, Mann-Whitney *p* = 0.0002
- Parieto-Occipital: *d* = −0.540, *t*-test *p* = 0.0008, Mann-Whitney *p* = 0.0002
- Parietal: *d* = −0.481, *t*-test *p* = 0.0031, Mann-Whitney *p* < 0.0001
- Temporal: *d* = −0.476, *t*-test *p* = 0.0031, Mann-Whitney *p* < 0.0001
- Frontal: *d* = −0.365, *t*-test *p* = 0.0320, Mann-Whitney *p* = 0.0009
- Central: *d* = −0.354, *t*-test *p* = 0.0351, Mann-Whitney *p* = 0.0002
- Occipital: *d* = −0.343, *t*-test *p* = 0.0420, Mann-Whitney *p* = 0.0009
- Fronto-Central: *d* = −0.268, *t*-test *p* = 0.1287, Mann-Whitney *p* = 0.0004

####  ACW_e showed the most attenuated regional differences

- Centro-Parietal: *d* = −0.513, *t*-test *p* < 0.0001, Mann-Whitney *p* = 0.0049
- Parietal: *d* = −0.401, *t*-test *p* = 0.0030, Mann-Whitney *p* = 0.0151
- Parieto-Occipital: *d* = −0.391, *t*-test *p* = 0.0038, Mann-Whitney *p* = 0.0184
- Temporal: *d* = −0.374, *t*-test *p* = 0.0088, Mann-Whitney *p* = 0.0067
- Central: *d* = −0.356, *t*-test *p* = 0.0073, Mann-Whitney *p* = 0.0415
- Frontal: *d* = −0.351, *t*-test *p* = 0.0086, Mann-Whitney *p* = 0.0320
- Fronto-Central: *d* = −0.334, *t*-test *p* = 0.0119, Mann-Whitney *p* = 0.0643
- Occipital: *d* = −0.310, *t*-test *p* = 0.0299, Mann-Whitney *p* = 0.0421

####  ACW_50 demonstrated more uniform regional effects

- Centro-Parietal: *d* = −0.431, *t*-test *p* = 0.0003, Mann-Whitney *p* < 0.0001
- Fronto-Central: *d* = −0.401, *t*-test *p* = 0.0012, Mann-Whitney *p* = 0.0002
- Central: *d* = −0.368, *t*-test *p* = 0.0026, Mann-Whitney *p* = 0.0030
- Parietal: *d* = −0.366, *t*-test *p* = 0.0052, Mann-Whitney *p* = 0.0004
- Frontal: *d* = −0.320, *t*-test *p* = 0.0178, Mann-Whitney *p* < 0.0001
- Parieto-Occipital: *d* = −0.307, *t*-test *p* = 0.0190, Mann-Whitney *p* = 0.0032
- Temporal: *d* = −0.275, *t*-test *p* = 0.0574, Mann-Whitney *p* < 0.0001
- Occipital: *d* = −0.266, *t*-test *p* = 0.0466, Mann-Whitney *p* = 0.0003

### Channel-wise analysis with multiple comparison correction

**Fig. 3:**
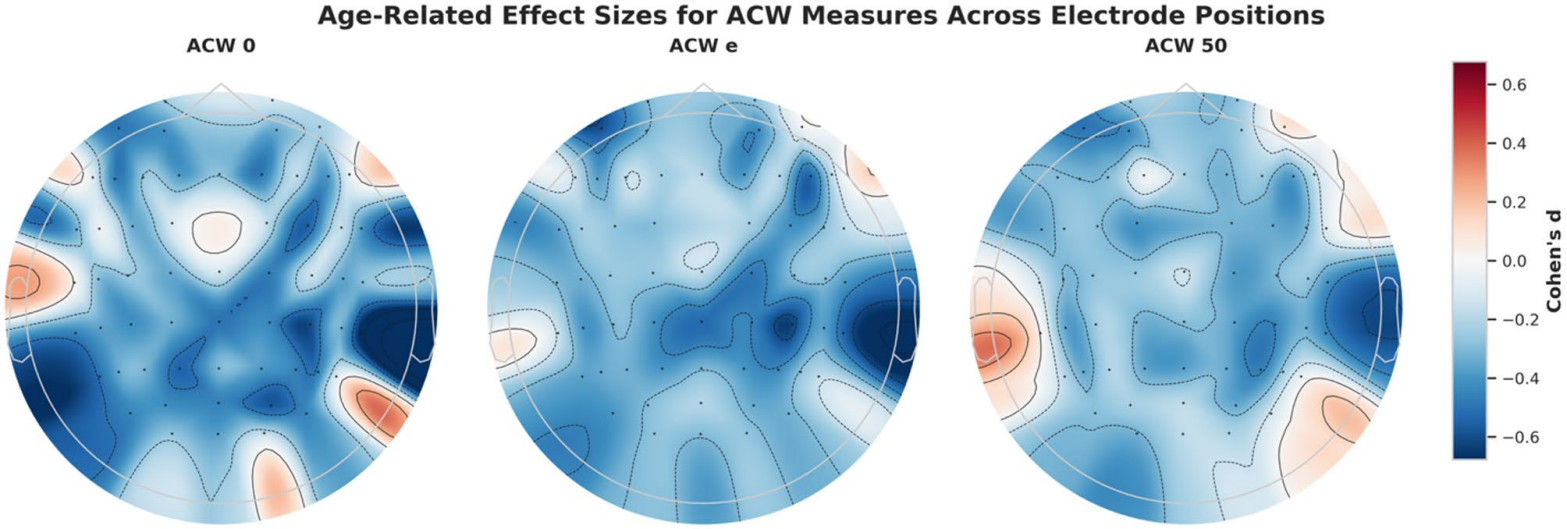
Age-related effect sizes for ACW measures across electrode positions. Topographic maps showing Cohen’s *d* effect sizes comparing young versus older adults across individual electrode positions. (A) ACW_0 demonstrates strongest effects in centro-parietal (CP4: *d* = −0.614) and parieto-occipital (PO4: *d* = −0.582) regions, following a posterior-to-anterior gradient. (B) ACW_e displays strongest centro-parietal effects (CP4: *d* = −0.631) with secondary parietal (P4: *d* = −0.439) effects, representing a similar posterior-to-anterior pattern. (C) ACW_50 shows strongest centro-parietal effects (CP4: *d* = −0.445) with fronto-central prominence (FC1: *d* = −0.406), representing a different spatial pattern. Color scale represents standardized effect sizes, with cooler colors indicating larger age-related decreases in older adults compared to young adults

Individual electrode analysis employed false discovery rate (FDR) correction using the Benjamini-Hochberg procedure (*α* = 0.05) to control for multiple comparisons across all available channels, applied separately to both parametric and non-parametric tests. The spatial distribution of these age-related effects is shown in Fig. 3, with distinct patterns emerging across the three ACW measures.

#### Significant channels by ACW measure (All electrodes Strategy)

Parametric Tests (*t*-test, FDR-corrected):

- ACW_0: 24/61 channels survived FDR correction (39.3% of channels).
- ACW_e: 28/61 channels survived FDR correction (45.9% of channels).
- ACW_50: 33/61 channels survived FDR correction (54.1% of channels).

Non-parametric Tests (Mann-Whitney *U*, FDR-corrected):

- ACW_0: 55/61 channels survived FDR correction (90.2% of channels).
- ACW_e: 23/61 channels survived FDR correction (37.7% of channels).
- ACW_50: 61/61 channels survived FDR correction (100.0% of channels).

#### Strongest Individual Channel Effects (Top 5, ranked by effect size magnitude, significant in both parametric and non-parametric tests)

ACW_0:

1. TP8: *d* = −0.678, 95% CI [−1.008, −0.348], *t*-test *p* < 0.0001, Mann-Whitney *p* < 0.0001
2. CP4: *d* = −0.614, 95% CI [−0.939, −0.289], *t*-test *p* = 0.0010, Mann-Whitney *p* < 0.0001
3. PO4: *d* = −0.582, 95% CI [−0.905, −0.259], *t*-test *p* = 0.0034, Mann-Whitney *p* < 0.0001
4. FC4: *d* = −0.560, 95% CI [−0.882, −0.238], *t*-test *p* = 0.0059, Mann-Whitney *p* < 0.0001
5. P1: *d* = −0.543, 95% CI [−0.864, −0.222], *t*-test *p* = 0.0059, Mann-Whitney *p* < 0.0001

ACW_e:

1. CP4: *d* = −0.631, 95% CI [−0.951, −0.311], *t*-test *p* < 0.0001, Mann-Whitney *p* = 0.0061
2. AF7: *d* = −0.571, 95% CI [−0.886, −0.256], *t*-test *p* < 0.0001, Mann-Whitney *p* = 0.0153
3. F6: *d* = −0.557, 95% CI [−0.885, −0.228], *t*-test *p* < 0.0001, Mann-Whitney *p* = 0.0113
4. CPz: *d* = −0.524, 95% CI [−0.824, −0.202], *t*-test *p* = 0.0010, Mann-Whitney *p* = 0.0153
5. TP8: *d* = −0.516, 95% CI [−0.831, −0.201], *t*-test *p* = 0.0005, Mann-Whitney *p* = 0.0172

ACW_50:

1. AF7: *d* = −0.486, 95% CI [−0.799, −0.172], *t*-test *p* = 0.0012, Mann-Whitney *p* < 0.0001
2. P4: *d* = −0.466, 95% CI [−0.781, −0.152], *t*-test *p* = 0.0012, Mann-Whitney *p* < 0.0001
3. CP4: *d* = −0.445, 95% CI [−0.741, −0.121], *t*-test *p* = 0.0012, Mann-Whitney *p* < 0.0001
4. TP8: *d* = −0.421, 95% CI [−0.736, −0.106], *t*-test *p* = 0.0026, Mann-Whitney *p* < 0.0001
5. AF3: *d* = −0.420, 95% CI [−0.730, −0.110], *t*-test *p* = 0.0047, Mann-Whitney *p* < 0.0001

Hemispheric pattern: Right-lateralized channels demonstrated the strongest effects, with centro-parietal and temporo-parietal regions being particularly prominent, suggesting potential hemispheric asymmetries in age-related INTs.

### Global versus regional network organization

#### Principal component analysis

Principal component analysis was conducted to differentiate between global brain-wide and regionally-specific age effects. The first principal component (PC1) captured a dominant proportion of variance across all ACW measures and electrode strategies

#### PC1 variance explained and age effects across strategies

**Table:** 

Strategy	ACW_0	ACW_e	ACW_50
All Electrodes (61 channels)	61.0% (*d* = −0.577, *p* = 0.009)	70.3% (*d* = −0.509, *p* = 0.005)	55.0% (*d* = −0.425, *p* = 0.018)
Empirical 95% (57 channels)	60.4% (*d* = −0.509, *p* = 0.012)	67.7% (*d* = −0.434, *p* = 0.007)	52.6% (*d* = −0.387, *p* = 0.014)
Empirical 100% (21 channels)	58.6% (*d* = −0.477, *p* = 0.004)	66.0% (*d* = −0.383, *p* = 0.005)	54.2% (*d* = −0.356, *p* = 0.006)

Both parametric (*t*-test) and non-parametric (Mann-Whitney *U*) tests confirmed statistical significance across all strategies, demonstrating that findings were not dependent on electrode inclusion criteria.

The exceptionally high variance explained by PC1 (55–70% across measures and strategies) indicates that age-related ACW differences represent a dominant, coordinated pattern of brain-wide variation rather than isolated regional effects. Secondary components (PC2-PC5) accounted for substantially smaller variance proportions (1.7–6.9%) and demonstrated no significant age effects (all *p* > 0.05), confirming the global nature of age-related ACW differences.

### Multi-level analysis validation

#### Cross-Level effect consistency

Age effects were systematically validated across three hierarchical levels of analysis to ensure robustness across different data aggregation approaches:

#### Effect size consistency across analysis levels (All electrodes Strategy)

**Table:** 

ACW Measure	Participant-Level	Segment-Level	Channel-Level	Consistency
ACW_0	*d* = −0.44, *p* = 0.008 (*n* = 196)	*d* = −0.45, *p* < 0.001 (*n* = 1,490)	*d* = −0.34, *p* < 0.001 (*n* = 12,199)	Consistent
ACW_e	*d* = −0.40, *p* = 0.002 (*n* = 196)	*d* = −0.43, *p* < 0.001 (*n* = 1,490)	*d* = −0.32, *p* < 0.001 (*n* = 12,199)	Consistent
ACW_50	*d* = −0.37, *p* = 0.003 (*n* = 196)	*d* = −0.37, *p* < 0.001 (*n* = 1,490)	*d* = −0.26, *p* < 0.001 (*n* = 12,199)	Consistent

Both parametric and non-parametric tests confirmed significance across all analysis levels, with effect sizes demonstrating systematic attenuation from participant-level to channel-level analysis.

Effect sizes demonstrated systematic attenuation from participant-level to channel-level analysis (average reduction ≈ 0.10 Cohen’s *d* units), reflecting the expected statistical dependency structure inherent in nested data. All cross-level comparisons were classified as “Consistent” across electrode strategies.

### Within-Group age relationship analysis

To distinguish between categorical and continuous aging patterns, age-ACW correlations were examined separately within young (20–35 years) and older (59–77 years) adult groups using both parametric (Pearson) and non-parametric (Spearman) approaches.

#### Young adults (*n* = 137)

No significant correlations emerged between age and any ACW measure after FDR correction (all p_FDR > 0.27).

#### Older adults (*n* = 59)

No significant correlations emerged between age and any ACW measure after FDR correction (all p_FDR > 0.76).

### Summary of principal findings

The present investigation yielded eight primary findings regarding age-related differences in ACWs:

1. Robust Age Group Differences: Young adults demonstrated significantly longer ACW values than older adults across all temporal measures (Cohen’s *d* = −0.328 to −0.480, all *p* ≤ 0.014), with both parametric and non-parametric tests yielding consistent results.
2. Cross-Strategy Robustness: Age effects replicated across three independent electrode selection approaches, with effect sizes varying by less than 0.05 units across strategies for most measures, confirming that the findings were not dependent on electrode inclusion criteria.
3. Methodologically-Informed Hierarchical Structure: Variance decomposition revealed minimal segment-level variance (2.5–3.5% across ACW measures), empirically supporting simplified analytical approaches focused on participant-level differences rather than complex multilevel modeling.
4. Global Network Organization: Principal component analysis revealed that age differences reflect brain-wide coordination patterns rather than regionally-specific effects, with PC1 explaining 55–70% of variance across strategies.
5. Systematic Spatial Heterogeneity: Despite global organization, effect magnitudes followed a posterior-to-anterior gradient, with the strongest effects in parieto-occipital regions and evidence of right-hemispheric bias.
6. Multiple Comparison Robustness: Individual electrode effects survived rigorous FDR correction, with 24–33 channels (39–54%) demonstrating significant age differences per ACW measure.
7. Absence of Within-Group Age Correlations: Within-group analyses revealed no significant age-ACW correlations in either young or older adults. However, given the restricted age ranges within each group (20–35 and 59–77 years), these null findings likely reflect limited statistical power to detect age-related slopes rather than evidence for categorical aging. Without middle-aged participants (36–58 years), we could not distinguish between categorical and continuous aging trajectories.
8. Multi-Level Consistency: Age effects demonstrated systematic patterns across participant, segment, and channel levels of analysis, confirming robustness across different data aggregation approaches.

The present investigation yielded consistent evidence for significant age-related reductions in ACW measures across all electrode selection strategies, with effects demonstrating global rather than regionally-specific patterns.

## Discussion

### Methodological validation and robust age effects

The present investigation addressed methodological concerns in EEG aging research while providing comprehensive evidence for age-related differences in INTs. Following the original LEMON study design, we treated age as a categorical variable comparing young and older adult groups. The empirical variance decomposition provided strong justification for simplified analytical approaches, with segment-level variance contributing minimally (2.5–3.5%) across all ACW measures, supporting participant-level focused analyses over complex three-level hierarchical modeling.

The consistency of findings across electrode selection strategies provides evidence for robustness in ACW aging research. Effect sizes remained stable whether analyzing all available channels (61) or the most conservative selection criteria (21 channels), with maximum variations of only 0.044 Cohen’s *d* units. This cross-strategy validation demonstrates that age-related differences in INTs are not artifacts of data completeness or electrode selection criteria, but represent genuine neurobiological phenomena detectable even under stringent data quality constraints.

### Interpretation of ACW findings

The consistent observation that young adults exhibit higher ACW values than older adults across all three temporal measures (Cohen’s *d* = −0.328 to −0.480) supports our hypothesis that ACWs decrease with age. Given that ACW quantifies the persistence of temporal correlations in neural activity [7], the higher values in young adults indicate that spontaneous brain activity maintains coordinated patterns over longer timescales compared to older adults.

Although we interpret shorter ACWs in older adults as reflecting changes in temporal integration capacity, alternative mechanisms warrant consideration. First, age-related increases in neural noise or reductions in signal-to-noise ratio could lead to faster decorrelation of neural signals without necessarily reflecting changes in functional temporal integration. Second, alterations in excitatory-inhibitory balance could drive shorter timescales by shifting the operating point of neural circuits. Third, the observed patterns might reflect compensatory processes, where older adults’ brains adopt different temporal processing strategies to maintain function despite structural decline.

The autocorrelation function is mathematically related to the power spectrum via the Wiener-Khinchin theorem. Age-related spectral changes (such as slowing of alpha peak frequency, altered frequency band power distributions, and changes in aperiodic 1/***f*** slope) could contribute to or even fully account for observed ACW differences. Without analyses controlling for spectral properties, we cannot determine whether ACW captures unique temporal dynamics beyond what is already reflected in traditional spectral measures.

### Global network organization and spatial patterns

The principal component analysis results provide evidence that age-related ACW differences primarily reflect global, brain-wide organizational changes rather than isolated regional modifications. The high variance explained by PC1 (55–70% across measures and strategies) indicates that aging affects INTs through coordinated network-level reorganization encompassing the entire cortical mantle. This global pattern was consistent across all electrode selection approaches.

This global pattern suggests that age-related changes in INTs may reflect fundamental alterations in brain network architecture, potentially involving modifications to synaptic strength distributions or large-scale connectivity patterns that coordinate activity across distributed brain regions [4, 8]. The absence of significant age effects in secondary components (PC2-PC5) further supports that the dominant pattern is brain-wide rather than compensatory recruitment of alternative networks, consistent with Northoff’s framework of inner time as a unified neural process [7, 8]. However, regional variations exist within this global pattern, as detailed below.

### Regional heterogeneity within global patterns

Despite the predominant global organization, spatial patterns varied by measure. ACW_0 showed a posterior-to-anterior gradient with the strongest effects in the parieto-occipital and centro-parietal regions, while ACW_50 and ACW_e demonstrated more distributed patterns including both frontal and posterior sites.

Right-hemisphere electrodes TP8 and CP4 showed strong effects across ACW measures, with substantial effect sizes (TP8: *d* = −0.678). This pattern may reflect differential aging rates between hemispheres or hemispheric specialization for temporal processing mechanisms that become more pronounced with advancing age.

This measure-specific heterogeneity in spatial patterns may reflect several possible mechanisms: (1) differential sensitivity of ACW measures to distinct underlying neural processes, (2) regional differences in the properties of neural oscillations and their age-related changes, or (3) heterogeneous aging trajectories across different cortical regions.

The concentration of strongest age effects in posterior regions (centro-parietal, parieto-occipital) contrasts with traditional frontal aging models [2]. This posterior emphasis may reflect several mechanisms. First, age-related changes in posterior alpha oscillations could particularly affect ACW measures in these regions. Second, the posterior-to-anterior gradient observed for ACW_0 and ACW_e suggests that different cortical regions exhibit distinct aging trajectories, with posterior sensory integration networks showing different temporal dynamics than frontal executive regions.

### Channel-wise analysis and multiple comparison robustness

Individual electrode analysis with rigorous FDR correction revealed substantial proportions of channels with significant age differences: 24/61 channels (39.3%) for ACW_0, 28/61 channels (45.9%) for ACW_e, and 33/61 channels (54.1%) for ACW_50. The survival of these effects after multiple comparison correction underscores the robustness and widespread nature of age-related changes in INT.

The strongest individual channel effects were concentrated in the temporo-parietal and parietal regions, with the most robust findings including TP8 (*d* = −0.678), CP4 (*d* = −0.614), and PO4 (*d* = −0.582) for ACW_0. These effect sizes represent medium-to-large magnitude differences according to Cohen’s conventions, indicating that specific brain regions show particularly pronounced age-related changes in temporal integration capacity.

### Cross-level consistency

Age effects were consistent across participant, segment, and channel levels of analysis, with effect sizes showing expected attenuation from participant-level (*d* ≈ −0.40) to channel-level (*d* ≈ −0.30) analysis and remaining statistically significant at all levels. This cross-level consistency confirms the reliability of age effects while reflecting the expected statistical dependency structure inherent in nested data.

### Clinical implications

The robustness of the findings across multiple validation approaches suggests that ACW measures may have potential utility as neurobiological markers of brain aging, though several critical steps remain before clinical application. Electrode selection consistency indicates that ACW measures remain viable even with limited channel coverage, which has practical implications for clinical settings. The global effects revealed by PCA suggest these measures could provide sensitive indices of brain-wide network integrity. However, the functional implications remain unclear without validation against cognitive and behavioral outcomes.

### Limitations and future directions

This analysis followed the original LEMON age design, which intentionally excluded middle-aged participants (36–58 years), creating a 22-year gap between young and older groups [15]. This gap prevents determining whether ACW changes follow categorical (step-wise) or continuous (gradual) trajectories across the lifespan. The restricted age ranges within each group (20–35 and 59–77 years) limited statistical power to detect within-group age correlations. The cross-sectional design cannot distinguish true aging effects from cohort effects [2]. Longitudinal studies tracking individual participants across multiple decades would provide definitive evidence regarding the timing and nature of transitions in INTs, while allowing examination of individual differences in aging trajectories and their relationship to self-experience and well-being.

ACW values are sensitive to preprocessing parameters including sampling rate, filtering, and epoch length. The use of 250 Hz downsampled data, 1–45 Hz bandpass filtering, and 60-second epochs may bias absolute ACW magnitudes and inter-measure relationships, particularly for ACW-0. The analysis relied on the LEMON team’s preprocessing pipeline without additional quality control steps beyond the original protocol. While the consistency of findings across multiple validation approaches argues against major artifact contamination, residual artifacts could potentially influence INT measures.

The choice of FCz as the EEG reference electrode could bias spatial distributions of effects. While FDR correction was applied for multiple comparisons, spatial dependence among electrodes (due to volume conduction and correlated sensors) could inflate discovery rates. Future investigations would benefit from parameter sensitivity analyses, alternative reference schemes (e.g., average reference, linked mastoids), and cluster-based permutation or spatial random-field corrections.

This study did not examine several potential moderators of age effects, including sex, health status, and lifestyle factors. Future work should report ICA artifact component counts, blink/ECG components, and vigilance proxies (e.g., alpha power) by age group.

Cognitive, behavioral, or clinical measures were not examined, limiting interpretation of functional significance. Without empirical validation against functional outcomes, it cannot be determined whether ACW differences have meaningful consequences for cognition, perception, or daily functioning. The relationship between ACW measures and spectral properties (e.g., alpha frequency, band power, spectral slope) was not examined, leaving uncertain whether ACW captures unique temporal dynamics beyond traditional spectral measures.

Future research priorities should include: (1) comprehensive longitudinal designs with balanced age distributions across the entire adult lifespan, particularly including middle-aged participants, to distinguish categorical from continuous aging trajectories and examine individual differences in aging patterns and their relationship to self-experience and well-being; (2) investigation of ACW changes during active cognitive tasks to examine task-dependent temporal dynamics and establish links to behavioral performance; (3) integration with validated measures of cognitive function to establish functional significance and biomarker utility, particularly tasks requiring temporal integration (e.g., working memory, sensory integration, temporal prediction); (4) systematic examination of the relationship between ACW and spectral properties (e.g., alpha peak frequency, band power, spectral slope) to determine whether these measures provide complementary or redundant information about age-related neural changes; (5) parameter sensitivity analyses across preprocessing choices (e.g., sampling rate, filtering parameters, epoch length) to establish optimal pipelines for ACW measurement; (6) examination of potential moderators of age effects including sex, health status, medication use, lifestyle factors (e.g., education, physical activity, cognitive engagement), and vigilance states; and (7) exploration of how cultural conceptions of self and aging influence temporal neural dynamics.

## Conclusion

This investigation provides robust evidence for age group differences in intrinsic neural timescales, with young adults demonstrating significantly longer ACWs than older adults across three measures. However, the functional significance of these differences requires validation through cognitive and behavioral measures, and the trajectory of change across the lifespan remains to be characterized through studies including middle-aged adults.

The results reveal that age-related changes in INTs reflect global brain-wide reorganization rather than isolated regional modifications, with effects demonstrating a posterior-to-anterior gradient for ACW_0 and ACW_e measures and evidence of right-hemispheric prominence in some regions. These patterns suggest that aging involves coordinated changes in temporal dynamics across distributed neural networks.

Beyond basic research into aging, these findings offer potential applications for understanding psychopathology, developing brain-age-appropriate interventions, and advancing theories of embodied cognition and temporal continuity in self-experience [7, 8, 16]. The methodological framework developed here provides tools for future investigations into the neural basis of temporal integration in aging, health, and disease.

## Data Availability

The datasets analyzed during the current study are publicly available in the LEMON repository: https://doi.org/10.1038/sdata.2018.308. The preprocessing pipeline and analysis code used in this study are available from the corresponding author on reasonable request.

## Abbreviations

ACW: Autocorrelation Window
AIC: Akaike Information Criterion
BIC: Bayesian Information Criterion
CMS: Cortical Midline Structures
EEG: Electroencephalography
FDR: False Discovery Rate
ICA: Independent Component Analysis
ICC: Intraclass Correlation Coefficient
INT: Intrinsic Neural Timescales
LEMON: Leipzig Study for Mind-Body-Emotion Interactions
PACC: Perigenual Anterior Cingulate Cortex
PC: Principal Component
PCA: Principal Component Analysis
PCC: Posterior Cingulate Cortex
